# Metabolomic Signature of Visceral Adiposity: Insights from a Population-Based Cohort

**DOI:** 10.3390/metabo16050343

**Published:** 2026-05-19

**Authors:** Khaled Naja, Najeha Anwardeen, Shamma Almuraikhy, Mohamed A. Elrayess, Ahmed Malki

**Affiliations:** 1 Biomedical Research Center, QU Health, Qatar University, Doha P.O. Box 2713, Qatar; khaled.naja@qu.edu.qa (K.N.); n.anwardeen@qu.edu.qa (N.A.); salmuraikhy@qu.edu.qa (S.A.); 2 College of Medicine, QU Health, Qatar University, Doha P.O. Box 2713, Qatar; 3 Biomedical Science Department, College of Health Sciences, QU Health, Qatar University, Doha P.O. Box 2713, Qatar

**Keywords:** visceral adiposity, untargeted metabolomics, 4-hyroxyglutamate, branched-chain amino acids, sphingomyelins

## Abstract

**Background:** Visceral adipose tissue (VAT) is a key determinant of cardiometabolic risk, yet its underlying molecular mechanisms remain incompletely characterized. Metabolomics offers an opportunity to identify circulating biomarkers that capture VAT-related biology beyond conventional clinical measures. **Methods**: We conducted a cross-sectional analysis of 2526 participants from the Qatar Biobank using untargeted serum metabolomics profiling. VAT was quantified using DXA-derived estimates and analyzed both as a continuous variable and by comparing individuals in the highest quartile to the remainder quartiles. Associations between metabolites and VAT were assessed using multivariate partial least squares discriminant analysis and adjusted linear regression models controlling for age, sex, and BMI, with Bonferroni correction for multiple testing. **Results**: Continuous VAT was associated with 106 metabolites, while the Q4 versus Q1–Q3 contrast identified 23 metabolites, with overlapping metabolites defining a robust core VAT signature. Higher VAT was characterized by coordinated elevation of branched-chain amino acids and their keto/hydroxy acid derivatives, glutamate, and central carbon intermediates, consistent with impaired mitochondrial oxidative decarboxylation. We further identified 4-hydroxyglutamate as a novel collagen-derived metabolite positively associated with VAT, suggesting a potential link between extracellular matrix remodeling and glutamate-centered metabolism. Additionally, greater VAT was associated with lower concentrations of glycine and glycine conjugates and reduced levels of unsaturated sphingomyelins and plasmalogens. **Conclusions**: These findings provide potential mechanistic insights into VAT-related metabolic dysfunction and identify candidate circulating biomarkers that may enable non-invasive assessment of visceral fat-associated cardiometabolic risk. Longitudinal and mechanistic studies are warranted to establish causality and clinical utility.

## 1. Introduction

Visceral adipose tissue (VAT) is a metabolically active fat depot located within the abdominal cavity, surrounding internal organs such as the liver, intestines, and pancreas. Unlike subcutaneous fat, VAT is strongly linked to the metabolic syndrome and cardiovascular events, even after adjustment for overall obesity [1]. VAT is a critical phenotype for understanding heterogeneous risk among people who are overweight or obese, and also among those with a seemingly normal body mass index, in whom excess visceral fat may still confer substantial adverse health effects [2]. VAT research remains an important area of study. The global burden of obesity and type 2 diabetes continues to rise, and VAT appears to be a more specific predictor of adverse outcomes than traditional anthropometric measures such as BMI or waist circumference. Moreover, VAT is modifiable but not easily measured in routine clinical practice, and gold standard imaging methods are costly, involve radiation exposure in the case of CT, or are not widely available. This underscores the need for surrogate biomarkers that capture VAT-related biology and can be deployed for risk stratification in large populations. Additionally, VAT-associated risk is not fully accounted for by standard lipids, glucose, or inflammatory markers, suggesting that key biological pathways remain incompletely characterized. Elucidating the molecular underpinnings of VAT could therefore improve the early detection of high-risk individuals and help refine preventive and therapeutic strategies.

Metabolomics provides a powerful means to elucidate the biochemical consequences of increased visceral adiposity. Investigating VAT through a metabolomics framework remains a timely and valuable approach for advancing the precision prevention and management of cardiometabolic disease. Our objective is to achieve a comprehensive characterization of the metabolic signature associated with visceral adiposity. In this cross-sectional study, we applied non-targeted serum metabolomics to examine metabolic profiles across the full VAT distribution and to identify metabolite patterns linked to both continuous VAT measures and individuals in the highest VAT quartile. This dual analytical strategy enables the identification of a robust core set of VAT-associated metabolites that remain consistent across different exposure definitions and analytical models, offering deeper insight into the molecular pathways that connect visceral fat accumulation with cardiometabolic risk. We hypothesize that higher DXA-derived visceral adiposity would be associated with a distinct circulating metabolomic profile that remains evident after adjustment for BMI.

## 2. Methods

### 2.1. Data Source and Study Participants

This study utilized data from the Qatar Biobank (QBB), a prospective population-based study that looked at a population sample of Qatari nationals and long-term residents of Qatar [3]. The data collection included a comprehensive socio-demographic questionnaire and various clinical parameters [4]. A subset of QBB data with clinical parameters and measured metabolomics was chosen (*n* = 2526). Our study included a sample of 1331 males and 1195 females. This research was approved by the Qatar Biobank’s institutional review boards (QF-QBB-RES-ACC-00178).

### 2.2. VAT and Clinical Measurements

All clinical measurements were performed at the Hamad Medical Corporation’s central laboratory, which is certified by the College of American Pathologists. Additionally, the dataset included information on medication usage [5], medical history, and a metabolomics profile covering more than 1000 metabolites using the Metabolon platform [6]. A full-body General Electric Lunar DXA scan was used to measure body composition (GE Healthcare, Madison, WI, USA). Participants were instructed to wear a light robe and no jewelry, and to lay flat and still on the scanning table. VAT was estimated from the DXA scan using GE Lunar CoreScan Software (version 14.0, GE Healthcare, Madison, WI, USA). VAT measures used in the subsequent analyses are derived from these DXA-based CoreScan estimates. VAT estimates from CoreScan were shown to be comparable to those derived from the gold standard MRI or CT scan in a previous validation study conducted in a subset of the same cohort [7].

### 2.3. Metabolomics

All participant serum samples were subjected to untargeted metabolomics using established protocols by Metabolon [8] using their standardized platform, and all metabolites reported were identified at Metabolomics Standards Initiative level 1 (confirmed structure by reference standard). Metabolite measurement was performed using a Thermo Scientific Q-Exactive high-resolution/accurate mass spectrometer (Thermo Fisher Scientific, Inc., Waltham, MA, USA) interfaced with a heated electrospray ionization (HESI-II) source and Orbitrap mass analyzer operated at 35,000 mass resolution along with Waters ACQUITY ultra-performance liquid chromatography (UPLC) (Waters Corporation, Milford, MA, USA). A thorough explanation of the process has already been provided [8]. Hits were matched with pre-existing library entries of over 3300 pure standard chemicals to identify the compounds. Compounds were divided into several groups according to their sources. Internal standards and quality checks were previously published [9]. In short, to adjust for discrepancies in sample preparation and instrument performance, a combination of stable isotope-labeled chemicals was utilized as internal standards. The stability and repeatability of the procedure were tracked over time using quality control samples. To reduce variability and guarantee the integrity of the samples, a systematic methodology was employed for pre-analytical sample management, including sample collection, storage, and preparation.

### 2.4. Statistical Analysis

VAT values were log-transformed to reduce right skewness and subsequently standardized within sex to generate sex-specific z-scores. VAT was analyzed primarily as a continuous exposure. For descriptive and confirmatory secondary analyses, VAT was additionally categorized into sex-specific quartiles, with the highest quartile (Q4) contrasted against the remaining participants (Q1–Q3). Metabolomics data were pre-processed according to standard quality control procedures. Metabolite features were log-transformed prior to statistical analysis. Principal component analysis (PCA) was performed on the metabolomics dataset, and the first two principal components (PC1 and PC2) were included as covariates in regression models to account for technical variations. Multivariate analysis was performed using partial least squares discriminant analysis (PLS-DA). Model performance was evaluated using M-fold cross-validation with 200 repeated permutations, and the Q^2^ values reported reflect this internal validation procedure, which was used to mitigate overfitting and provide a conservative estimate of predictive performance. Associations between visceral adiposity and individual metabolites were assessed using linear regression models, with metabolites as the dependent variable and VAT as the primary independent variable. All models were adjusted for age, sex, and body mass index (BMI). BMI adjustment is performed to evaluate whether the identified associations reflect VAT-specific effects beyond general obesity. Regression coefficients represent the change in metabolite per one standard deviation increase in VAT. Similar regression models were also fitted for categorical VAT, comparing individuals in the highest VAT quartile (Q4) with those in the lower quartiles (Q1–Q3), adjusting for the same set of covariates. Multiple testing correction was performed using Bonferroni correction with the significant level set at 5.5 × 10^−5^. Bonferroni correction was applied as a stringent threshold to prioritize highly robust associations. Metabolites were mapped to pre-established metabolic pathways by Metabolon (Morrisville, NC, USA).

## 3. Results

### 3.1. General Characteristics of Participants

The study cohort consisted of 2526 participants, including 1195 females (47.4%) and 1331 males (52.6%). The median age of participants was 38 years (IQR:29–48). The mean age of the cohort was 39.1 years, indicating a predominantly adult population with balanced sex representation. The median BMI was 28.3 kg/m^2^ (IQR: 24.8–32.4 kg/m^2^), with a mean BMI of 28.9 kg/m^2^. Individuals in the highest VAT quartile were older than the rest of the cohort and therefore age was treated as a confounder and adjusted for in all regression models. Propensity score matching was not applied, as VAT represents a continuous biological exposure rather than a treatment assignment, and matching would unnecessarily reduce the sample size and distort the exposure contrast. Table 1 shows the participant characteristics stratified by VAT quartiles.

### 3.2. Molecular Patterns Associated with VAT

**Figure 1:**
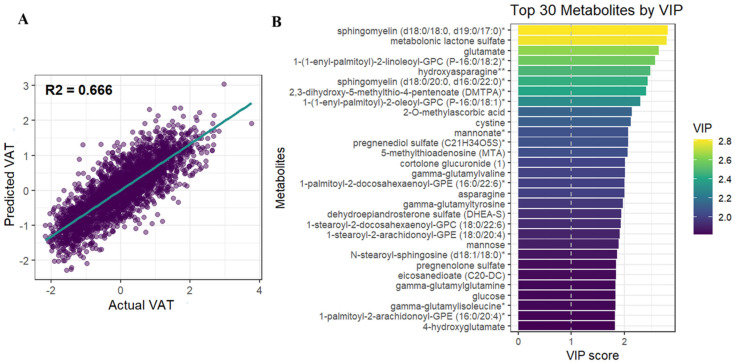
(**A**) The fitted regression line and cross-validated model performance (R^2^ = 0.66) show the link between observed DXA-derived VAT z-scores and PLS-predicted VAT z-scores based on the metabolomics data. (**B**) The PLS model’s variable importance in projection (VIP) scores were used to rank the top 30 metabolites; higher VIP values indicate a larger contribution to the metabolomic signature linked to VAT. Metabolites are colored by VIP score intensity (purple—low to yellow—high). * and ** indicates a compound that has not been officially confirmed based on a standard, but that Metabolon is confident in its identity.

A partial least squares analysis of metabolomics data was conducted to assess the molecular signature associated with VAT. The model demonstrated a good agreement between the predicted and observed values of VAT (cross-validated R^2^ = 0.66, Q^2^ = 0.41 and Pearson correlation of 0.82 between the observed and predicted VAT scores, *p* < 2.2 × 10^−6^), as shown in Figure 1A. Variable importance of projection (VIP) was retrieved from the model and the top metabolites contributing to the VAT signature are shown in Figure 1B.

### 3.3. Univariate Linear Regression

#### 3.3.1. VAT as Continuous Variable

**Figure 2:**
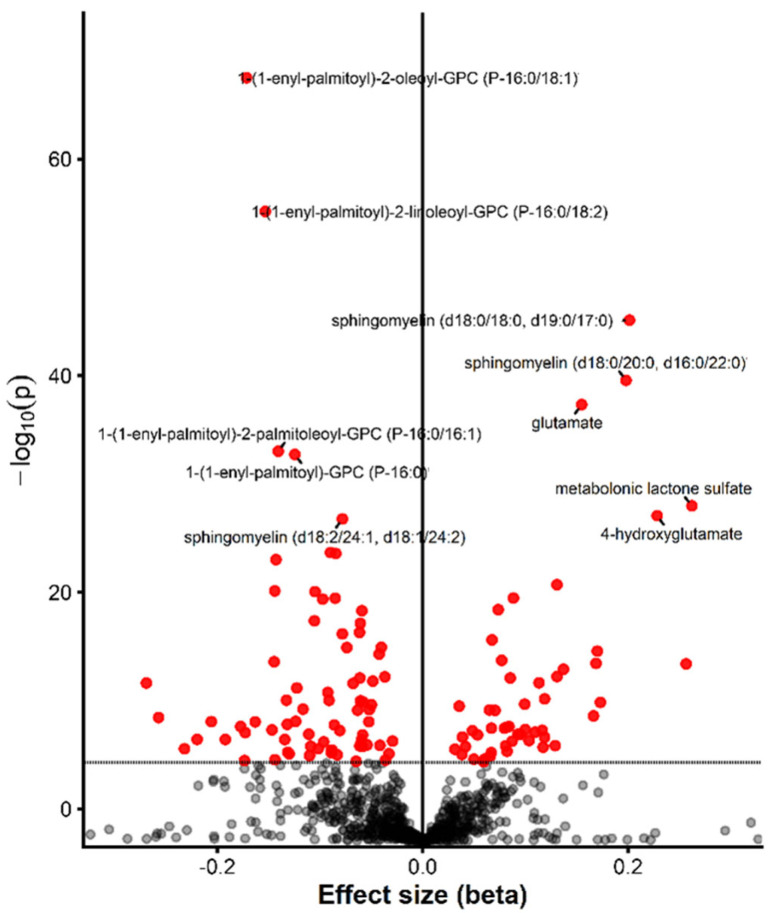
Volcano plot illustrating the results of univariate linear regression analyses assessing the association between individual metabolites and VAT. The x-axis represents the beta coefficient from the models and y-axis represents the −log10-transformed *p*-values. Bonferroni-significant metabolites are highlighted in red with top 10 most significant metabolites labeled.

To validate the multivariate findings and assess metabolite associations while accounting for potential confounding variables, univariate linear regression analyses were performed for each metabolite individually, adjusting for age, sex, and BMI. A total of 106 metabolites were associated with VAT at the Bonferroni-corrected significance threshold (<5.5 × 10^−5^). Figure 2 depicts the 10 most significant metabolites from the univariate analysis, and the full set of associated metabolites is provided in Supplementary Table S1. A substantial proportion of the metabolites identified as significant in the univariate models overlapped with those ranked highly in the VIP analysis, indicating strong agreement between the multivariate and univariate approaches.

#### 3.3.2. VAT as Categorical Variable

Similar univariate linear regression analyses were performed for each metabolite individually while adjusting for age, sex, and BMI to assess associations with the highest quartile (Q4) versus the other quartiles (Q1-Q3). A total of 23 metabolites were associated with Q4 at a Bonferroni level of significance (<5.5 × 10^−5^). Table 2 depicts the results from the univariate analysis.

## 4. Discussion

Visceral fat is a harmful type of fat that increases the risk of metabolic syndrome, type 2 diabetes, and cardiovascular disease, beyond what can be explained by body weight or total body fat alone. In this large, population-based cohort, we characterized the metabolomic signature of visceral adiposity using DXA-derived VAT and untargeted serum metabolomics. In our study, VAT was modeled both as a continuous trait and using a quartile-based comparison. In adjusted analyses, continuous VAT was associated with 106 metabolites at Bonferroni significance, whereas the Q4 versus Q1–Q3 contrast identified 23 metabolites. Importantly, several metabolites were consistently associated with VAT across both modeling strategies. These overlapping metabolites will constitute the main focus of our discussion, as they define a robust core set of VAT-associated metabolites supported by both exposures coding.

The first marked observation is the positive association of branched-chain amino acids (BCAAs) and many of their corresponding keto and hydroxy acid metabolites with higher VAT levels. This is in line with previous work [10,11,12] showing that visceral fat accumulation is accompanied by elevated circulating BCAAs, reflecting their impaired catabolism in adipose tissue and other peripheral organs. Indeed, in obesity and insulin resistance, downregulation of BCAA catabolic enzymes in visceral adipose tissue leads to reduced oxidative disposal of BCAAs [13], causing the accumulation of both the parent amino acids and their early catabolites in the circulation. This interpretation is reinforced by the higher glutamate concentrations observed at higher VAT levels, since BCAA transamination uses alpha-ketoglutarate as an amino group acceptor to generate glutamate together with the branched-chain α-keto acids. Of note, the increase in glutamate with higher VAT has been well documented [14,15]. In our data, all four components of this reaction (BCAA, alpha-ketoglutarate, glutamate and BCKA) increase with VAT, suggesting not a simple shift in equilibrium but a state of chronically increased transamination flux coupled with incomplete downstream oxidation.

Extending beyond this initial interpretation, our metabolomic results revealed that these BCAAs and related metabolites accumulate together with multiple central carbon intermediates, including pyruvate, α-ketoglutarate, and 2-oxoadipate, in association with VAT. Interestingly, these metabolites converge on a shared biochemical feature; their further catabolism depends on oxidative decarboxylation by structurally related mitochondrial α-keto acid dehydrogenase complexes [16], namely branched-chain keto acid dehydrogenase, pyruvate dehydrogenase, α-ketoglutarate dehydrogenase, and 2-oxoadipate dehydrogenase. This pattern is consistent with a systemic bottleneck at the level of mitochondrial oxidative decarboxylation capacity in individuals with higher visceral adiposity. In this context, impaired flux may arise from redox imbalance, lipid-induced metabolic inflexibility, or inhibitory post-translational regulation of dehydrogenase complexes [16,17,18]. Our results are also in line with proteomic and functional studies of visceral adipose tissue in obesity, which demonstrate coordinated alterations and downregulation of mitochondrial proteins [19] and increased the oxidative modification of complex II in VAT, indicating heightened redox stress and reduced oxidative capacity in the visceral depot. Taken together, our findings could support a potential model in which visceral adiposity is characterized by impaired mitochondrial oxidative disposal of diverse carbon substrates, moving the interpretation of elevated BCAAs away from increased input alone and toward defective oxidative flux as a key metabolic correlate of VAT-related metabolic dysfunction.

The second observation is the increase in 4-hydroxyglutamate with VAT. To our knowledge, 4-hydroxyglutamate has not previously been described in relation to adiposity and has only emerged as a stronger biomarker of pre-eclampsia [20,21], primary hyperoxaluria type 3 [22], and gestational diabetes mellitus [23]. Biochemically, 4-hydroxyglutamate can be converted to glutamate by 4-hydroxyglutamate aminotransferase, which may in part explain the elevated glutamate levels with VAT observed in our study. 4-hydroxyglutamate is formed from 4-hydroxyproline, which is derived almost exclusively from collagen turnover [20]. Surprisingly, collagen turnover in adipose tissue is closely linked to visceral adiposity [24]. As visceral fat expands, the extracellular matrix undergoes dynamic remodeling characterized by both increased collagen deposition and degradation [25,26,27]. This altered extracellular matrix remodeling shapes adipocyte function, promotes low-grade inflammation, and may contribute to the adverse metabolic profile associated with excess visceral fat [26]. Interestingly, pre-eclampsia has been shown to be associated with visceral adiposity in early pregnancy [28,29], which further supports the plausibility of its association with VAT in our cohort. Together, these considerations suggest that elevated 4-hydroxyglutamate in individuals with higher VAT could reflect a heightened collagen turnover within expanding visceral adipose tissue, and may represent a potential novel circulating biomarker of visceral adiposity that warrants replication and formal evaluation in independent cohorts.

A third observation was the inverse association of glycine and glycine conjugates with VAT. In agreement with our finding, lower circulating glycine has been previously linked to greater visceral adiposity and reduced insulin sensitivity [30,31]. N-acetylglycine has also been shown to be the top metabolite inversely associated with central and overall adiposity, with higher levels predicting lower fat mass across populations [32]. Cinnamoylglycine, a glycine conjugate and putative microbial metabolite, has been linked to lower levels of atherogenic lipids and better gut microbial diversity [33], and its negative association with VAT in our data is therefore consistent with a shift toward a less favorable, VAT-associated metabolic and microbial milieu. A further aspect of this third observation was the inverse association of sphingomyelins and plasmalogens with VAT. Our previous work [34,35] demonstrated that sphingomyelins were more abundant in individuals with a lower triglyceride–glucose index and lower fatty liver index, indicating an insulin-sensitive and less steatotic phenotype, thereby challenging the prevailing view that sphingolipids are uniformly deleterious. At the same time, in our cohort, the saturated dihydrosphingomyelins remained positively associated with higher VAT, indicating that different sphingomyelin subclasses and their saturation levels can distinguish metabolically healthier from more visceral fat-enriched phenotypes.

Our study has several notable strengths. Firstly, it leverages a large, well-characterized, population-based cohort with balanced sex representation and a broad age range, enhancing the statistical power. Secondly, we applied high-coverage, untargeted serum metabolomics and rigorous quality control procedures, enabling a comprehensive and high-quality characterization of circulating metabolites. Thirdly, VAT was analyzed both as a continuous exposure and in quartiles, and the findings were cross-validated using multivariate and univariate models, yielding a consistent core set of VAT-associated metabolites across analytical approaches. Importantly, all metabolite–VAT associations were adjusted for age, sex and BMI, helping to isolate VAT-specific metabolic signatures beyond general adiposity. Moreover, all samples were collected in the fasting state, which helps to reduce short-term dietary variability. Information on medication use was available but was not included as a covariate in the primary models. In the Qatar Biobank, participants use a wide range of cardiometabolic and non-cardiometabolic drugs with heterogeneous, and sometimes opposing, metabolic effects, which makes it unlikely that any single medication class alone explains the coherent VAT-associated signature observed in our study across multiple amino acid and lipid pathways. Nonetheless, we cannot entirely exclude confounding by medication use, and our findings should therefore be interpreted as VAT-associated metabolomic patterns in a real-world, treated population.

On the other hand, several limitations should be acknowledged. The cross-sectional design precludes causal inference, so we cannot determine whether the observed metabolomic alterations are causes, consequences, or only correlates of higher VAT; therefore, our pathway-level interpretations are hypothesis-generating and should not be taken as direct evidence of altered mitochondrial function or extracellular matrix remodeling. Our sample consists of Qatari nationals and long-term residents, which may limit generalizability to other ethnic and environmental backgrounds, and external validation in independent cohorts is needed. Although DXA-derived VAT estimates correlate well with CT/MRI, they remain an indirect measure and may not capture all aspects of visceral fat distribution or ectopic fat depots such as hepatic or epicardial fat. Finally, despite stringent multiple-testing correction and adjustment for BMI, residual confounding and measurement error cannot be entirely excluded, and therefore, we cannot definitively separate VAT-specific biology from broader cardiometabolic disturbance, and some of the observed associations may reflect general metabolic risk rather than visceral adiposity per se.

## 5. Conclusions

Our study provides a comprehensive characterization of the circulating metabolomic signature associated with visceral adiposity in a large, population-based cohort. Higher VAT was characterized by coordinated elevation of BCAA and derivatives, glutamate, and central carbon intermediates such as pyruvate, α-ketoglutarate and 2-oxoadipate, supporting a potential model of impaired mitochondrial oxidative decarboxylation capacity. We further highlight 4-hydroxyglutamate as a novel collagen-derived metabolite positively associated with VAT, potentially linking extracellular matrix remodeling to glutamate-centered metabolic disturbances. In parallel, greater VAT was associated with depletion of glycine and glycine conjugates and with lower levels of unsaturated sphingomyelins and plasmalogens. Future longitudinal and mechanistic studies are required to establish whether the VAT-associated metabolomic signature we describe can predict cardiometabolic events, to evaluate its clinical and prognostic utility as a biomarker of VAT-related risk, and to clarify the causal pathways by which visceral adiposity influences systemic metabolism.

## Figures and Tables

**Table 1:** Demographic characteristics of participants stratified by Q4 vs. Q1-Q3 of VAT scores.

Test	Variable	Q1-Q3 (N = 1896)	Q4 (N = 630)	*p* Value
VAT z-score		−0.36 (−1.014–0.15)	1.21 (0.96–1.54)	<0.0001
General characteristics	Gender (M/F)	999/897	332/298	0.99
	Age	34 (28–44)	49 (40–56)	<0.0001
	BMI (kg/m^2^)	26.8 (23.9–29.8)	33.8 (30.5–37.2)	<0.0001
	Systolic blood pressure (mmHg)	111 (102–120)	122 (114–132)	<0.0001
	Diastolic blood pressure (mmHg)	71 (65–78)	78 (72–85)	<0.0001
Glycemic profile	Fasting blood glucose (mmol/L)	5 (4.7–5.4)	5.6 (5.0125–7)	<0.0001
	HbA_1C_ (%)	5.4 (5.1–5.6)	5.9 (5.5–6.5)	0.199
	C-peptide (ng/mL)	2.07 (1.5–3.05)	3.09 (2.34–4.56)	<0.0001
	Insulin (uU/mL)	8.7 (6–14.8)	15.1 (10.1–27.1)	<0.0001
Lipid profile	Total cholesterol (mmol/L)	4.8 (4.3–5.4475)	5.1 (4.4925–5.7)	<0.0001
	HDL cholesterol (mmol/L)	1.33 (1.12–1.59)	1.17 (0.99–1.39)	<0.0001
	LDL cholesterol (mmol/L)	3 (2.34–3.46)	3.2 (2.49–3.7)	<0.0001
	Triglyceride (mmol/L)	1.06 (0.79–1.5)	1.5 (1.1–2.1)	<0.0001
Kidney function	Creatinine (µmol/L)	67 (56–79)	68 (57–78)	0.503
	Urea (mmol/L)	4.3 (3.5–5.1)	4.5 (3.7–5.3)	<0.0001
	Bicarbonate (mmol/L)	26 (25–28)	26 (24–29)	0.78
	Total protein (g/L)	73 (71–76)	73 (70.25–76)	0.435
Cardiac function	NT-proBNP (pg/mL)	24 (12.9–39)	25 (12.5–46.9)	0.207
	Homocysteine (µmol/L)	8.3 (6.9–10.1)	8.5 (7.1–10.3)	0.289
Liver function	ALT (U/L)	18 (13–27)	23 (17–33)	<0.0001
	AST (U/L)	18 (15–21.75)	19 (16–23)	<0.0001
	GGT (U/L)	18 (12–29)	28 (19–40)	<0.0001
	SHBG (nmol/L)	43 (29–64)	36.3 (26–50.175)	<0.0001

Data are expressed as mean (standard deviation) or median (interquartile range), depending on the distribution assessed using the Shapiro–Wilk normality test. Abbreviations: BMI, body mass index; HbA1C, glycated hemoglobin; HDL, high-density lipoprotein; LDL, low-density lipoprotein; NT-proBNP, N-terminal pro-B-type natriuretic peptide; ALT, alanine transaminase; AST, aspartate aminotransferase; GGT, Gamma-glutamyl transferase; SHBG, sex hormone-binding globulin.

**Table 2:** Linear regression analysis to determine the metabolites associated with VAT Q4.

Metabolite	Super-Pathway	Sub-Pathway	Estimate	*p*-Value	Bonferroni
3-methyl-2-oxobutyrate	Amino Acid	Leucine, Isoleucine and Valine Metabolism	0.10	7.6 × 10^−14^	6.9 × 10^−11^
Pyruvate	Carbohydrate	Glycolysis, Gluconeogenesis, and Pyruvate Metabolism	0.09	1.4 × 10^−12^	1.3 × 10^−9^
Glutamate	Amino Acid	Glutamate Metabolism	0.14	4.5 × 10^−12^	4.1 × 10^−9^
3-methyl-2-oxovalerate	Amino Acid	Leucine, Isoleucine and Valine Metabolism	0.11	1.1 × 10^−11^	9.9 × 10^−9^
4-hydroxyglutamate	Amino Acid	Glutamate Metabolism	0.23	2.5 × 10^−11^	2.3 × 10^−8^
Alpha-hydroxyisovalerate	Amino Acid	Leucine, Isoleucine and Valine Metabolism	0.16	7.3 × 10^−11^	6.6 × 10^−8^
1-palmitoyl-2-palmitoleoyl-GPC (16:0/16:1)	Lipid	Phosphatidylcholine	0.18	2.8 × 10^−10^	2.5 × 10^−7^
Glucose	Carbohydrate	Glycolysis, Gluconeogenesis, and Pyruvate Metabolism	0.08	9.0 × 10^−10^	8.1 × 10^−7^
2-oxoadipate	Amino Acid	Lysine Metabolism	0.13	9.6 × 10^−10^	8.7 × 10^−7^
Xanthine	Nucleotide	Purine Metabolism	0.15	2.2 × 10^−9^	2.0 × 10^−6^
Sphingomyelin (d18:2/24:1, d18:1/24:2)	Lipid	Sphingomyelins	−0.08	2.9 × 10^−9^	2.6 × 10^−6^
Glycine	Amino Acid	Glycine, Serine and Threonine Metabolism	−0.07	3.2 × 10^−9^	2.9 × 10^−6^
Sphingomyelin (d18:0/18:0, d19:0/17:0)	Lipid	Dihydrosphingomyelins	0.15	3.9 × 10 ^−9^	3.5 × 10^−6^
1-(1-enyl-palmitoyl)-2-oleoyl-GPC (P-16:0/18:1)	Lipid	Plasmalogen	−0.11	5.7 × 10^−9^	5.1 × 10^−6^
Taurolithocholate 3-sulfate	Lipid	Secondary Bile Acid Metabolism	−0.33	7.3 × 10^−9^	6.7 × 10^−6^
N-acetylglycine	Amino Acid	Glycine, Serine and Threonine Metabolism	−0.14	1.1 × 10^−8^	9.9 × 10^−6^
Alpha-ketoglutarate	Energy	TCA Cycle	0.12	1.2 × 10^−8^	1.1 × 10^−5^
2-hydroxy-3-methylvalerate	Amino Acid	Leucine, Isoleucine and Valine Metabolism	0.14	1.5 × 10^−8^	1.3 × 10^−5^
Sphingomyelin (d18:2/24:2)	Lipid	Sphingomyelins	−0.09	1.6 × 10^−8^	1.4 × 10^−5^
Valine	Amino Acid	Leucine, Isoleucine and Valine Metabolism	0.06	3.1 × 10^−8^	2.8 × 10^−5^
Leucine	Amino Acid	Leucine, Isoleucine and Valine Metabolism	0.08	4.7 × 10^−8^	4.2 × 10^−5^
Isoleucine	Amino Acid	Leucine, Isoleucine and Valine Metabolism	0.05	4.8 × 10^−8^	4.3 × 10^−5^
Cinnamoylglycine	Xenobiotics	Food Component/Plant	−0.35	4.9 × 10^−8^	4.4 × 10^−5^

## Data Availability

The datasets used and/or analyzed during the current study are available from the corresponding author on reasonable request.

## Supplementary Materials

The following supporting information can be downloaded at: https://www.mdpi.com/article/10.3390/metabo16050343/s1, Table S1: Linear regression analysis to determine the metabolites associated with VAT, while adjusting for age, sex, BMI, and principal components 1 and 2.
